# Globular adiponectin inhibits osteoblastic differentiation of vascular smooth muscle cells through the PI3K/AKT and Wnt/β-catenin pathway

**DOI:** 10.1007/s10735-021-10012-2

**Published:** 2021-08-16

**Authors:** Yun Zhou, Li-Long Wei, Rui-Ping Zhang, Cheng-Wu Han, Yongtong Cao

**Affiliations:** Laboratory Medicine, China-Japan Friendship Hospital, No.2 Yinghua Street, Chaoyang District, Beijing, 100029 China

**Keywords:** Globular adiponectin, Osteoblastic differentiation, Vascular smooth muscle cells, PI3K/AKT and Wnt/β-catenin pathway

## Abstract

Lipid metabolism is closely related to the improvement of vascular calcification (VC) in chronic kidney disease (CKD). Globular adiponectin (gAd) has been reported to be involved in the development of VC in CKD, but the detailed regulatory role remains unclear. The present study is aimed to investigate the biological function and the underlying regulation mechanism of gAd in the process of VC during CKD. Vascular smooth muscle cells (VSMCs) calcification was determined by Alizarin Red S staining. Protein signaling related with VC was tested by western blotting. The expression and intracellular localization of runt-related transcription factor 2 (Runx2) was detected by immunofluorescence and uraemic rat with VC was established by a two-step nephrectomy. Combined with the results of Alizarin Red S staining, we discovered that β-glycerophosphate (β-Gp)-induced the osteoblastic differentiation of VSMCs was significantly reversed by gAd treatment. Along with the VSMCs calcification and the increase of Runx2 in β-Gp-exposed VSMCs, the activities of protein kinase B (AKT) and Wnt/β-catenin pathway were enhanced, but that were counteracted by the exposure of gAd in rat and human VSMCs. After administration with agonists of the Wnt (SKL2001) and AKT (SC79), there appeared more osteoblastic differentiation and higher expression of Runx2 in gAd-treated VSMCs, but showing lower impact in the presence of SC79 than that in the presence of SKL2001. In the in vivo experiments, intravenous injection of gAd also significantly inhibited VC and Runx2 level in uraemic rat in a dose-dependent manner, possibly through regulating Wnt/β-catenin pathway. This study demonstrates that gAd ameliorates osteoblastic differentiation of VSMCs possibly by blocking PI3K/AKT and Wnt/β-catenin signaling transduction. The findings provide an important foundation for gAd in treating VC in kidney diseases.

**Supplementary Information:**

The online version contains supplementary material available at 10.1007/s10735-021-10012-2.

## Introduction

Cardiovascular diseases (CVD) are the leading cause of disease mortality worldwide (Franz et al. 2010). Of note, patients with uremia often suffer from CVD (Adamczak et al. 2009). Overall, compared with the general population, there is approximately 50-fold increase of CVD mortality rate in uremia dialysis patients (Hou 2005), accounting for nearly 44.2–51.0% of overall mortality (Liu et al. 2014). Although vitamin K antagonists, direct oral anticoagulants, warfarin, heparin, parathyroid hormone and vitamin D compounds are used in chronic kidney disease (CKD) treatment (Harel et al. 2014; Aursulesei and Costache 2019; Palmer et al. 2007; Chen et al. 2018), there are still many challenges remaining for treating vascular calcification (VC) in uremic patients. Therefore, it is necessary to explore new potential drug to relieve VC in uremia patients.

Increased cardiovascular morbidity and mortality in uremia dialysis patients is associated with VC (Bellasi and Raggi 2012). In uremic patients, calcification of coronary artery and vascular tunica media contribute to the increased cardiovascular mortality. Artery calcification in uremic patients induces cardiovascular stiffening, leading to systolic hypertension, left ventricular hypertrophy and reduced coronary perfusion (London 2013). Although several biomarkers, such as calcium, phosphate and alkaline phosphatase play important roles in pathological pathways of VC in CKD (Alderson et al. 2013; Nascimento et al. 2014; Qureshi et al. 2015; Golembiewska et al. 2020), the exact pathogenic mechanisms remain unclear. It is confirmed that VC is associated with abnormal lipid metabolism. A decrease in high-density lipoprotein (HDL) cholesterol and an increase in low-density lipoprotein (LDL) cholesterol promote the process of VC (Ketteler et al. 2017). Sevelamer can improve VC in uremic patients by reducing lipid levels (Brandenburg et al. 2009; Wang et al. 2015). It suggests that targeting lipid metabolism possibly has become a promising anti-VC strategy in kidney disease, especially uremic patients.

Adiponectin, a key component in lipid metabolic process, can down-regulate the expression of the osteogenic transcription factor osterix by inhibiting the activation and nuclear transport of signal transducer and activator of transcription 3 (STAT3), resulting in the reduction of VC (Lu et al. 2019). Adiponectin also inhibits VC through reducing alkaline phosphatase (ALP), runt-related transcription factor 2 (Runx2), and bone morphogenetic protein 2 (BMP-2) (Lu et al. 2015). Of note, plasma adiponectin level in uremic patients is two to three times higher than healthy individual (Martinez Cantarin et al. 2014). Increased mortality in CKD patients is associated with high adiponectin level and high adiponectin has been used as a predictor for CKD progression in men. It implies that abnormal adiponectin level may be involved in the progression of CKD (Menon et al. 2006; Kollerits et al. 2007). Additionally, adiponectin can regulate lipid metabolism in mammals. Adiponectin promotes the synthesis, oxidation and transport of fatty acids (Ji et al. 2020). Meanwhile, adiponectin activates peroxisome proliferators activated receptor γ (PPARγ) through the interaction between adiponectin receptors (AdipoRs)-adaptor protein phosphotyrosine PH domain and leucine zipper 1 (APPL1), resulting in the regulation of the fatty acids synthesis and lipid metabolism (Holland et al. 2011; Kovalchuk et al. 2003; Mao et al. 2006; Yamauchi et al. 2014). These data demonstrate that adiponectin plays a crucial role in regulating vascular lipid metabolism and CKD.

In native conditions, adiponectin exists in the form of polymer, such as low molecular weight (LMW) trimer (67 kDa), middle molecular weight (MMW) hexamer (140 kDa), and high molecular weight (HMW) multimer (300 kDa) contains more than 18 monomers which are complex by full length adiponectin (Achari and Jain 2017). Full length adiponectin contains a NH2-terminal hyper-variable region, a COOH-terminal C1q-like globular domain, and a collagenous domain consisting of 22 Gly-XY repeats in the middle. Globular adiponectin (gAd) is mentioned as the COOH-terminal C1q-like globular domain, which is biologically active (Fruebis et al. 2001). There are three main adiponectin receptors, AdipoR1, AdipoR2 and T-cadherin. AdipoR1 is widely expressed in human tissue, and mainly in VC. AdipoR1 has high affinity with gAd and low affinity with full length adiponectin. AdipoR2 mainly expressed in the liver and recognizes the full-length adiponectin (Yamauchi et al. 2003). T-cadherin only recognizes hexamer and multimer of adiponectin (Hug et al. 2004). There was a study showed that gAd reduces VC via inhibition of ER-stress-mediated smooth muscle cell apoptosis (Lu et al. 2015), and no further researches focused on mechanism of gAd in VC regulation.

Given that abnormal vascular lipid metabolism in CKD can result in VC, we speculated that adiponectin might be involved in the progression of VC in uremic patients.

Herein, we demonstrated that gAd inhibited osteoblastic differentiation of vascular smooth muscle cells (VSMCs) in vitro and in vivo. Furthermore, we explored the molecular mechanism of gAd in inhibiting VSMCs calcification. This study implicated the protentional clinical significance of gAd supplement in suppressing VC in uremic patients. gAd may be one of the potential candidates for the improvement of VC and VC-associated diseases.

## Materials and methods

### Isolation and identification of VSMCs

Six-week-old male Sprague–Dawley (SD) rats were purchased from Shanghai Xipuer–Bikai Laboratory Animal Co., Ltd. All animal procedures performed in this study were approved by the Institutional Animal Care Committee of China-Japan Friendship Hospital.

SD rats were sacrificed by cervical dislocation. The abdominal aorta was taken out using sterilized instruments from rats after they were sterilized using 75% alcohol. And then the abdominal aorta was placed into phosphate-buffered saline (PBS). The intima and adventitia of abdominal aorta were stripped carefully, the artery was cut into small pieces. Then, these artery pieces were placed in a centrifuge tube contained with 0.2% collagenase I (C5894, Sigma). After incubation in a CO_2_ incubator (SW-CJ-1FD, Thermo Scientific) for 2 h, two times volume of DMEM medium (1552510, Gibco) was added to stop the digestion. Then the cell suspension was centrifuged for 5 min, and the pellet was resuspended in DMEM medium. Finally, the cell suspension was filtered through a 200-mesh filter once, and cells were disseminated into 10-cm cell culture dish. VSMCs were cultured in DMEM medium supplemented with 10% fetal bovine serum (FBS, 10099-141, Gibco) at 37 °C containing 5% CO_2_. DMEM medium was changed every 3 days. After three passages, the morphology of VSMCs were observed by an optical microscope (Olympus BX53). The purity of VSMCs were identified by α-SMA immunofluorescence staining. VSMCs were harvested after digestion and centrifuge. Then, VSMCs were cultured with 100 uL DMEM medium at 5 × 10^4^/mL concentration in 96-well plate. VSMCs were fixed in 100 uL 4% paraformaldehyde for 15 min at room temperature (RT). Then, cells were washed three times with PBS, followed by incubation in blocking solution for 1 h at RT. The cells were incubated in anti-α-SMA overnight at 4℃, washed with PBS, and incubated in FITC-labeled mouse anti-rabbit fluorescent secondary antibody for 1 h at RT. Then, cells were washed three times with PBS and incubated with DAPI. Finally, the expression of α-SMA in VSMCs were examined by laser scanning confocal microscope (Nikon C2).

T/G HA-VSMC cell line was obtained from ATCC. Cells were cultured in F12K medium (21127030, Gibco) supplemented with 10% FBS at 37 °C containing 5% CO_2_.

### Cell treatment

β-glycerol phosphate (β-Gp, HY-D0886, MCE) was used to induce VSMCs calcification and H/G HA-VSMCs. Briefly, cells were divided into control group, model group and gAd treatment group. Cells in model group were treated with β-Gp (10 mM, HY-D0886, MCE) for 48 h. For the co-treatment with β-Gp and gAd, cells were treated with β-Gp (10 mM) and gAd (1 μg/mL, 044481, US Biological) for 48 h. β-Gp and gAd were dissolved in DMSO, and isometric DMSO was added to the control group cells. For the exposure to agonists of Wnt and AKT, SKL2001 (20 μM, HY-101085, MCE), an activator of Wnt, and SC79 (4 μg/mL, HY-18749, MCE), an activator of AKT, were co-treated with β-Gp and gAd for 48 h in VSMCs. Cells were harvested for the subsequent experiments.

### Alizarin Red S staining

Alizarin Red S staining was performed in VSMCs after 14 days of culture. Firstly, 1% alizarin red S dye solution was prepared by using 0.1 M tris–Hcl 100 mL (pH 8.3) and 0.1 g alizarin red S (Xi’an Hat Biotechnology Co., Ltd). VSMCs were washed three times with PBS. Then, cells were fixed with formaldehyde at RT for 15 min. After stained with alizarin red S for 15 min at RT, cells were washed three times with PBS again. And cell morphology was observed and photographed under microscope (Olympus BX53).

For the staining of calcium nodules, the abdominal aortas were dissected from the rats and cut into a piece of 0.5 cm in length. The rat aortic tissue was firstly dehydrated with gradient alcohol (75% alcohol for 4 h, 85% alcohol for 2 h, 90% alcohol for 1.5 h, 95% alcohol for 1 h, absolute ethanol I for 0.5 h and absolute ethanol II for 0.5 h). After alcohol dehydration, the tissue block was hyalinized using the mixture of anhydrous ethanol and xylene (1:1) for 10 min. Then, the transparent tissues were embedded in paraffin using Leica CM3050 cryostat. For the Alizarin Red S staining, paraffin-embedded specimens were dewaxed using the following procedure: xylene I (10 min), xylene II (10 min), absolute ethanol I (5 min), absolute ethanol II (5 min), 95% alcohol (3 min), 90% alcohol (3 min), 80% alcohol (2 min), 70% alcohol (2 min). Then, the sections were rinsed with distilled water for 2 min. The paraffin-embedded tissue was used to make 5 μm-thickness pathological section. After staining in 1% Alizarin red S at RT for 5 min, the sections were rinsed with distilled water and the staining results were observed by the microscope. Calcium-positive cells were appeared in orange-red.

### Western blot

Total proteins were extracted from VSMCs or the aorta tissues according to the manufacturer’s instructions. In brief, equal amounts of protein was separated by SDS-PAGE and transferred onto an activated PVDF membrane (Amersham Biosciences, Piscataway, NJ, USA) after electroblotting. After blocking with 5% non-fat milk for 2 h, the membrane was incubated with the primary antibodies overnight at 4 °C. The detailed information of primary antibodies were as follow: GADPH (ab181602, Abcam, 1:1000), phosphorylated-β-catenin (ab75777, Abcam, 1:500), β-catenin (ab32572, Abcam, 1:5000), Wnt inhibitory factor-1(WIF) (ab155101, Abcam, 1:2000), Runx2 (ab23981, Abcam, 1:1000), phosphorylated-AKT (ab81283, Abcam, 1:5000), Protein Kinase B (AKT) (ab188099, Abcam, 1:2000), receptor activator of NF-kB Ligand (RANKL) (ab239607, Abcam, 1:1000) and alkaline phosphatase (ALP) (ab228636, Abcam, 1:1000), phosphorylated-STAT3 (ab76315, Abcam, 1:2000), STAT3 (ab109085, Abcam, 1:1000), BMP2 (ab214821, Abcam, 1:1000). The membrane was rinsed with TBST three times (5 min/once), and then incubated at RT for 2 h with secondary antibody. After incubation, the membrane was rinsed three times with TBST (5 min/once). By using an enhanced chemiluminescence kit, protein bands were visualized by GeneGnome chemiluminescence imaging system.

### Immunofluorescence

VSMCs were plated in 96-well plate at a concentration of 5 × 10^4^/mL. Cells were harvested at indicated time points. Cells were washed with PBS three times and fixed with 100 μL 4% paraformaldehyde for 15 min at RT. Then, cells were permeabilized with 0.5% Triton X-100 in PBS for 20 min. Subsequently, cells were washed three times with PBS, followed by incubation in goat serum for 30 min at RT. After absorbing the blocking solution with absorbent paper, cells were incubated with anti-Runx2 primary antibodies (ab23981 Abcam, 1:1000) overnight at 4 °C. After primary antibody incubation, sections were washed three times with PBS and incubated with secondary antibody for 1 h at RT. Nuclei were counterstained with Hoechst (4082S, Cst, 1:1000) for 5 min at RT. The staining results were observed with fluorescence microscope (Leica).

### Animal models

6–8 weeks healthy SD male rats (200 ± 20 g) were used to establish uraemic rat with VC. All rats had free access to food and drinking water. Rats were maintained on a 12:12 h (light: dark) cycle at 20 °C room temperature environment. All experimental procedures were in accordance with the NIH Animal Care and Use Committee guidelines and approved by the Care of Experimental Animals Committee of China-Japan Friendship Hospital. After a week of adjustable feeding, rats were randomly divided into 4 groups: Sham group (sham operation group), model group (5/6 subtotal nephrectomy group), treatment group with low dose of drug (5/6 subtotal nephrectomy group with intravenous injection of 1 μg/kg gAd) and treatment group with high dose of drug (5/6 subtotal nephrectomy group with intravenous injection of 5 μg/kg gAd) (n = 8 per group). For the model establishment, rats underwent 5/6 subtotal nephrectomy with a two-step method. In brief, rats were anesthetized using ketamine at a dose of 100 mg/kg. The left kidney was exposed and the kidney capsule was peeled off. Then, 1/3 of the superior and inferior poles of the left kidney was removed. Seven days after the first stage operation, the second stage operation was performed. Under the general anesthesia, the right kidney was exposed. After ligating the kidney pedicle, the right kidney was removed. Rats were treatment with gAd at four weeks after operation.

### Statistical analysis

Several independent experiments were performed to guarantee reproducibility of findings. All data are presented as mean ± standard error of the mean (SEM). Data were analyzed using the software package SPSS 21.0. Student’s *t* test and was used to evaluate statistical differences between two groups. ANOVA test was used to evaluate statistical differences among more than two groups. *P* < 0.05 was considered statistically significant difference.

## Results

### gAd abolishes β-GP-induced osteogenic differentiation of rat VSMCs

**Fig. 1:**
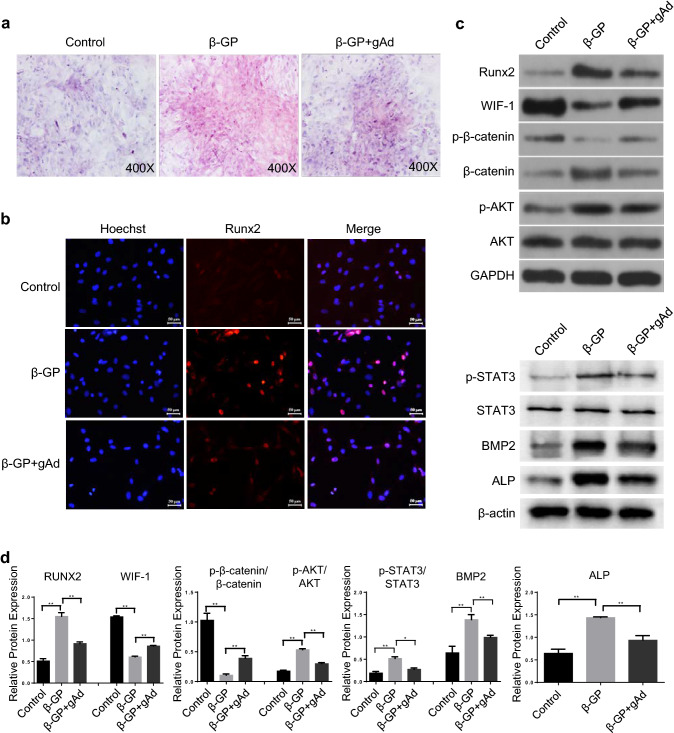
Effects of gAd on osteoblastic differentiation of rat VSMCs. **a** The level of osteoblastic differentiation of VSMCs was analyzed by Alizarin Red S Staining. **b** Detection of Runx2 subcellular localization using immunofluorescence. The nucleus was stained with Hoechst (blue) and Runx2 by antibody (red). Scale bar: 20 μm. **c** Western blot showed that the expression of WIF-1, phosphorylation-β-catenin, β-catenin, Runx2, phosphorylation-AKT, AKT, BMP2, ALP, phosphorylation-STAT3 and STAT3 protein in VSMCs treated by different chemical compound. **d** Quantitative analysis of protein expression of Runx2, WIF-1, BMP2, ALP, phosphorylation-β-catenin/β-catenin, phosphorylation-AKT/AKT, phosphorylation-STAT3/STAT3. The data are shown as the means ± SD. N = 3 per group.^*^P < 0.05, ^**^P < 0.01. *β-Gp* β-glycerol phosphate, *gAd* globular adiponectin. (Color figure online)

To investigate the specific effect of gAd on the osteoblastic differentiation of VSMCs, we firstly isolated VSMCs from rat aorta. Combined with the microscope imaging results, we observed that the morphology of arterial smooth muscle cells were normal, showing spindle shapes, or thin and long fusiform swirling patterns, or cobblestone appearances (Supplemental Fig. 1a). Additionally, utilizing the immunofluorescent staining, the proportion of α-SMA positive cells was close to 100% (Supplemental Fig. 1b). This result indicates that rat arterial smooth muscle cells were successfully isolated. Subsequently, we analyzed the function of gAd on VSMCs calcification by Alizarin red S staining. The binding of Alizarin Red S and calcium ions will generate a red complex, which can be used to analyze the deposition of orange-red calcium (Lievremont et al. 1982). Herein, compared with the control group, we observed an enhancement of the calcareous sediments and deposition of orange-red calcium of VSMCs in the β-GP treatment group. However, VSMCs calcification was notably reversed after gAd exposure (Fig. 1a). Moreover, we analyzed the expression of Runx2 which was a reliable marker for osteoblastic differentiation. Our results showed that β-GP significantly increased the expression of Runx2 and its distribution in the nucleus compared with un-treated cells. However, the expression of Runx2 in β-GP-induced VSMCs was significantly declined after gAd exposure (Fig. 1b). Meanwhile, western blotting results showed that the expression of Runx2 was increased significantly in model group compared with control group, whereas it was notably reversed in gAd treated model rat (Fig. 1c, d).

To explore the possible regulatory mechanism by which gAd ameliorated osteogenic differentiation, Wnt/β-catenin and PI3K/AKT signaling pathways were analyzed which were closely related with osteogenic differentiation (Liang et al. 2019; Ohashi et al. 2019). WIF-1 is an important negative regulator of Wnt/β-catenin signaling pathway (Tang et al. 2017). Herein, the results showed that β-GP-induced the decline of WIF-1 in VSMCs compared with the control group, which was restored in the presence of gAd. Along with the alteration of WIF-1, we observed that β-catenin phosphorylation was obviously repressed, while the expression of total β-catenin protein was up-regulated in calcific VSMCs induced by β-GP. When administrated with gAd, both β-GP-mediated decline of WIF-1 and activated Wnt/β-catenin signal cascades were abrogated in VSMCs (Fig. 1c, d). The data indicated that β-GP exposure might reactivate the Wnt/β-catenin pathway and gAd administration observably abolished the regulatory role of β-GP in VSMCs. In addition, we observed that the phosphorylated AKT in VSMCs of model group was up-regulated compare with control group. However, compared with model group, the activity of AKT, STAT3, BMP2 and alkaline phosphatase were significantly reversed in gAd treatment group. There has no significant change in total AKT and STAT3 among different groups (Fig. 1c, d). In summary, these data suggest that gAd may improve VC of VSMCs by inhibiting the activation of nuclear transcription factor Runx2 via Wnt/β-catenin and PI3K/AKT signaling pathway.

### gAd reverses β-GP-induced osteogenic differentiation of human VSMCs

To verify the role of gAd in β-GP-induced osteogenic differentiation of human VSMCs, we utilized a human VSMC cell line T/G HA-VSMC. The cells were separated into control group, β-GP group and β-GP plus gAd group. Immunofluorescence analysis of Hoechest and Runx2 indicated that β-GP plus gAd group showed decreased Hoechst compared with β-GP or control group (Supplemental Fig. 2a). Runx2 expression were the highest in β-GP group which indicated that Runx2 was induced by β-GP and reversed by gAd (Supplemental Fig. 2a). Wondering the mechanisms of gAd in human VSMCs differentiation, we measured the expression levels of Runx2, WIF-1, BMP2, ALP, AKT, STAT3 and β-catenin and phosphorylation of AKT, STAT3 and β-catenin. Data showed that β-GP can induced the expression of Runx2, β-catenin, BMP2, ALP, and p-AKT. β-GP also downregulated WIF-1 and the p-β-catenin expression. However, gAd recovered the induction of β-GP on human VSMCs (Supplemental Fig. 2b, c). Data above suggests that gAd may improve VC of VSMCs by inhibiting the activation of nuclear transcription factor Runx2 via Wnt/β-catenin and PI3K/AKT signaling pathways.

### Wnt agonist reverses the gAd-mediated improvement of osteoblastic differentiation of VSMCs

**Fig. 2:**
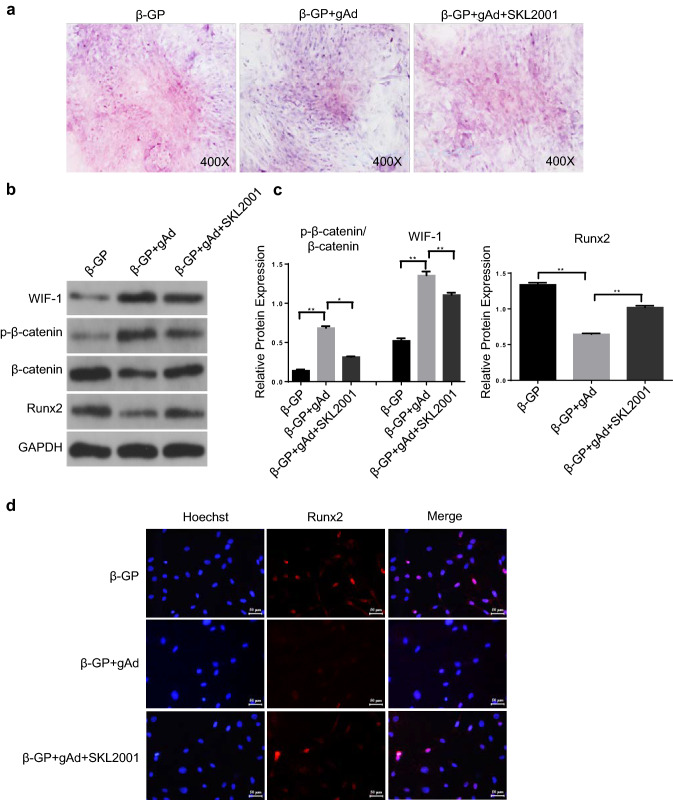
Role of Wnt agonist on gAd-initiated the improvement of osteoblastic differentiation in VSMCs. **a** Alizarin Red S Staining was used to detect the osteoblastic differentiation of VSMCs in different groups. **b** The protein levels of WIF-1, β-catenin phosphorylation, β-catenin, Runx2 were tested by western blotting. **c** Quantitative analysis of protein expression of Runx2, WIF-1 and phosphorylation-β-catenin/β-catenin. **d** Confocal images showing the localization of Runx2 in VSMCS. Runx2 (red), one of the important osteogenic genes. Nuclei: Hoechst, blue. Scale bar: 20 μm. The data are shown as the means ± SD. N = 3 per group.^*^P < 0.05, ^**^P < 0.01. *β-Gp* β-glycerol phosphate, *gAd* globular adiponectin, *SKL2001* an agonist of the Wnt. (Color figure online)

To confirm the effects of Wnt/β-catenin pathway on osteoblastic differentiation of VSMCs, SKL2001, an agonist of Wnt, was employed to stabilize the activation of nuclear β-catenin in VSMCs. In comparison to VSMCs treated with β-GP, we found that the mineralized bone matrix was obviously reduced after gAd treatment as shown by Alizarin Red S staining results. By contrast, the gAd-mediated decrease of osteoblastic differentiation in β-GP-induced VSMCs was restored after SKL2001 treatment (Fig. 2a). The levels of WIF-1 were robustly elevated after treatment with gAd in β-GP-exposed VSMCs; however, the gAd-mediated restoration of WIF-1 was impeded by the addition of Wnt agonist. Additionally, along with the destruction of Wnt/β-catenin pathway in β-GP-exposed VSMCs after treatment with gAd, the expression of Runx2 was also inhibited. However, after co-administration of SKL2001, the Wnt/β-catenin pathway was re-activated, and the expression of Runx2 was notably enhanced compared to β-GP and β-GP + gAd-treated VSMCs (Fig. 2b, c). Furthermore, gAd significantly inhibited the expression of nuclear Runx2 in β-GP-exposed VSMCs during osteoblast differentiation, whereas this effect was restored by the addition of SKL2001 (Fig. 2d). Our data indicate that Wnt/β-catenin pathway contributes to β-GP-induced osteogenic differentiation of VSMCs. gAd can inhibit osteoblastic differentiation of VSMCs possibly by inhibiting the activation of Wnt/β-catenin pathway.

### AKT activation impairs the gAd-mediated reduction of osteoblastic differentiation of VSMCs

**Fig. 3:**
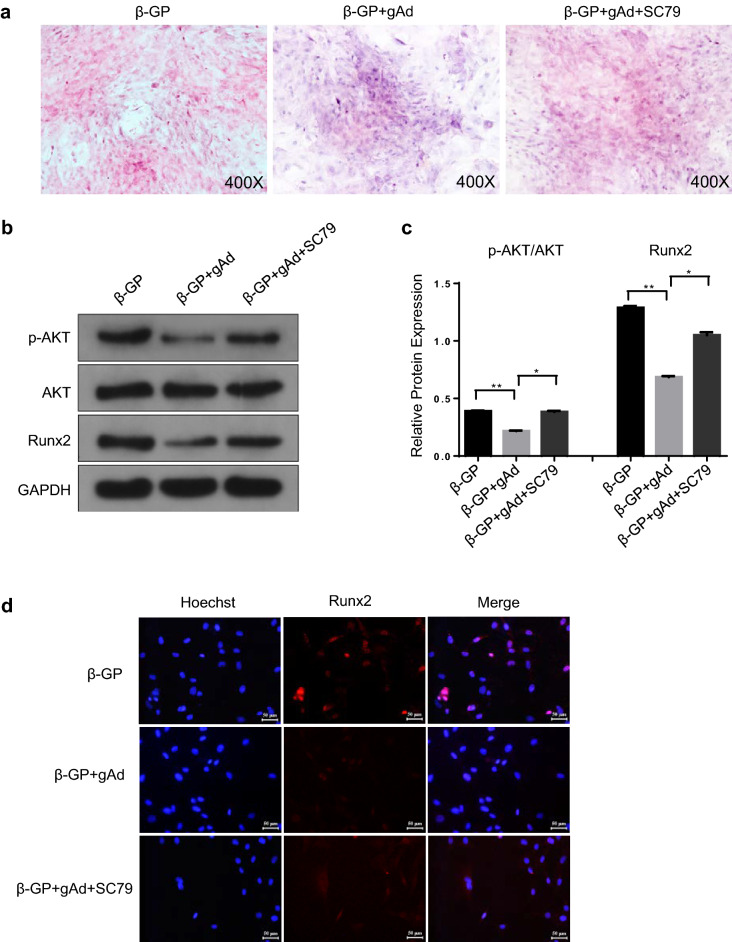
Role of AKT activator on gAd-initiated the improvement of osteoblastic differentiation in VSMCs. **a** Alizarin Red S Staining was performed in VSMCs after different chemical compound treatment for 48 h. **b** Western blotting analysis of the expression of phosphorylated AKT, AKT and Runx2 in VSMCs with or without gAd treatment. **c** Quantitative analysis of protein expression of Runx2 and phosphorylation-AKT/AKT. **d** Immunostaining showing the positioning and expression of Runx2. The data are shown as the means ± SD. N = 3 per group.^*^P < 0.05, ^**^P < 0.01. *β-Gp* β-glycerol phosphate, *gAd* globular adiponectin, *SC79* an activator of AKT. Runx2 (red); Nuclei (Hoechst, blue). Scale bar: 20 μm. (Color figure online)

**Fig. 4:**
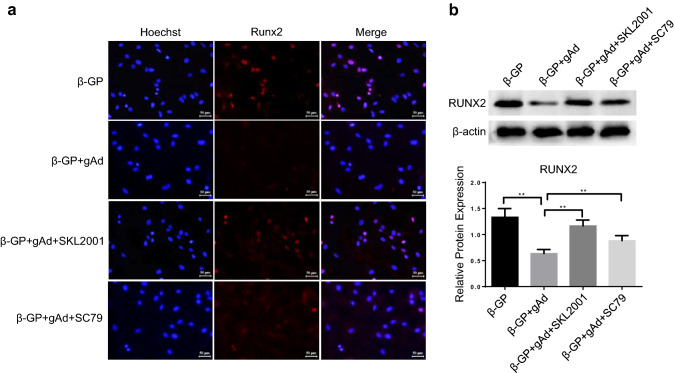
Role of AKT activator and Wnt agonist on gAd-initiated the improvement of osteoblastic differentiation in VSMCs. **a** Immunostaining showing the positioning and expression of Runx2. *Gp* β-glycerol phosphate, *gAd* globular adiponectin, *SC79* an activator of AKT, *SKL2001* an agonist of the Wnt. Runx2 (red); Nuclei (Hoechst, blue). Scale bar: 20 μm. **b** Quantitative analysis of protein expression of Runx2. The data are shown as the means ± SD. N = 3 per group. ^**^P < 0.01. (Color figure online)

Next, SC79, an AKT activator which can bind to the plecktrin homology domain of AKT, was used to identify the effects of PI3K/AKT pathway on osteoblastic differentiation of VSMCs. In accordance with the above results, gAd administration reversed the β-GP-induced calcification of VSMCs and the increase of Runx2 in nuclei (Fig. 3a–d). Besides, gAd also suppressed the activity of AKT in β-GP-induced VSMCs (Fig. 3b, c). However, we observed an increase of mineralized bone matrix in VSMCs after gAd treatment compared to gAd treatment alone in β-GP-induced VSMCs after SC79 treatment (Fig. 3a). Furthermore, the addition of SC79 re-activated the AKT signaling, followed with the moderate increase of Runx2 expression and nuclear activity of Runx2 in β-GP-induced VSMCs in the presence of gAd (Fig. 3b–d). To compare the function of SC97 with SKL2001 in gAd medicated reduction of VSMCs differentiation, we analyzed the Runx2 expression by immunofluorescence and western blot analysis (Fig. 4a, b), data showed that SC97 with SKL2001 both could promote Runx2 expression, and SKL2001 exhibited a stronger affection than SC97. However, AKT reactivation had a low impact on the restoration of Runx2. Therefore, gAd inhibits osteoblastic differentiation of VSMCs possibly independent of PI3K/AKT pathway.

### gAd injection ameliorates VC in a rat model of uremia

**Fig. 5:**
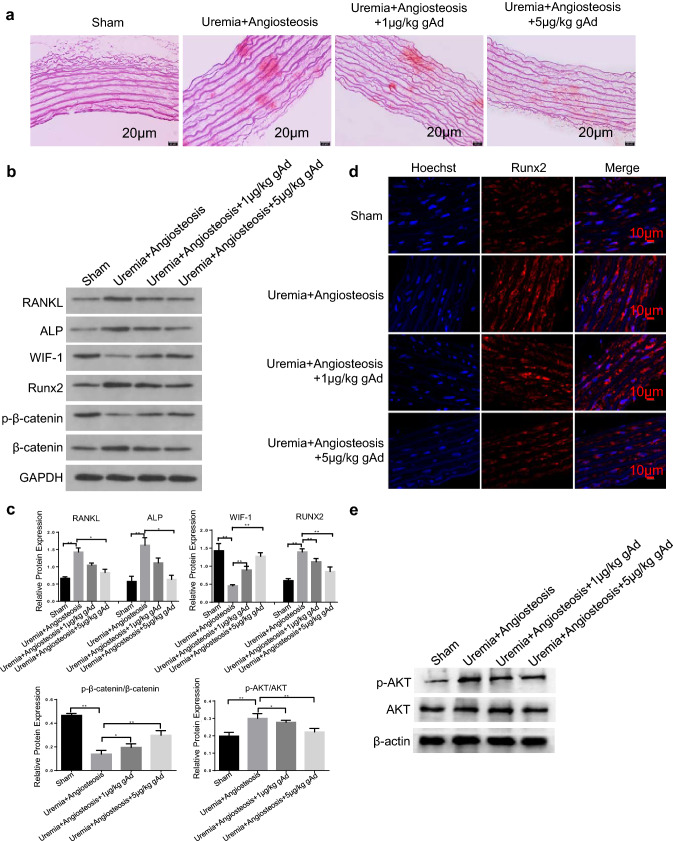
Effects of gAd on calcium nodules in a rat model of uremia. **a** Alizarin red staining was used to detect the calcium nodules in aortic vessels of rat. **b** Western blotting analysis of RANKL, ALP, WIF-1, phosphorylated β-catenin, β-catenin and Runx2 expression in different groups. **c** Quantitative analysis of protein expression of RANKL, ALP, Runx2, WIF-1, phosphorylation-AKT/AKT and phosphorylation-β-catenin/β-catenin. **d** The expression and localization of Runx2 in rat aortic vessels were detected by immunofluorescence. *gAd* globular adiponectin. Runx2 (red); Nuclei (Hoechst, blue). Scale bar: 20 μm. **e** Western blot analysis of AKT and phosphorylated AKT expression in different groups. The data are shown as the means ± SD. N = 3 per group.^*^P < 0.05, ^**^P < 0.01. (Color figure online)

Next, we evaluated whether gAd could inhibit calcium nodules in a rat model of uremia. We firstly constructed a rat model of uremia using nephrectomy. On the basis of results of Alizarin Red S staining, abundant mineral deposition, multiple small spots of calcification and more calcium nodules occurred in the uremia group compared with the sham group which indicated that the rat model of uremia with VC was successfully established. By contrast, the amount of calcium nodules was significantly reduced in the gAd-treated group compared to model group, showing even less in high-dose of gAd injection (5 μg/kg) (Fig. 5a). Thus, the gAd-improved VC of uremic rat was concentration dependent. Then, we analyzed the expression of reliable markers for osteoblastic differentiation, including RANKL, ALP and Runx2 by western blotting. The expression of RANKL, ALP and Runx2 was significantly increased in model group compared with sham group, whereas the phenomenon was significantly inhibited in the gAd-treated group in a dose-dependent manner (Fig. 5b, c). In addition, compared with the sham group, the expression of WIF-1 and phosphorylated β-catenin was significantly declined, the expression of total β-catenin was robustly elevated in model group, whereas the activation of Wnt/β-catenin pathway in aortic vessels of uremic rat was re-inactivated by gAd administration (Fig. 5b, c). Additionally, we also assessed the expression of nuclear Runx2 in the aortic vessels of different groups by immunofluorescence. Compared with the sham group, the Runx2 in the uremia group presented point accumulation, and the fluorescence intensity of Runx2 in the nucleus was significantly increased in uremic rats. However, gAd treatment notably reduced the fluorescence intensity of Runx2 in vivo (Fig. 5d). To confirm the activity of AKT, we examined the phosphorylation of AKT by western blot analysis. The activation of AKT was attenuated significantly by gAd (Fig. 5c, e). In summary, these data suggest that gAd may inhibit VC in a rat model of uremia by inhibiting transcription factor Runx2 via inhibiting Wnt/β-catenin pathway.

## Discussion

In the present study, we found that gAd could inhibit osteogenic differentiation of VSMCs possibly through targeting the PI3K/AKT and Wnt/β-catenin pathway. Accumulating evidence suggests that gAd inhibits the conversion of VSMCs to osteoblast-like cells, and slow down the development of cardiovascular tissue lesions (Proudfoot et al. 2001; Shroff et al. 2013). However, whether adiponectin could inhibit VC in uremic patients remain unclear. Therefore, the findings in the present study provides a new theoretical foundation for gAd in treating uremic patients with VC.

gAd inhibits VC of VSMCs through combining with the gAd receptor 1 (Luo et al. 2009). gAd also negatively regulate the VC of coronary artery (Okamoto et al. 2013). Consistent with previous studies, we found that gAd inhibited the osteogenic differentiation of VSMCs induced by β-GP. Runx2 plays a key role in the osteoblast cell differentiation and bone development (Sancisi et al. 2017). The expression of Runx2 is increased during the transformation of smooth muscle cells to the osteoblastic phenotype (Liberman et al. 2011). Furthermore, downregulation of Runx2 prevented the VC of VSMCs (Liu et al. 2019b). These studies indicate that the elevation of Runx2 can be served as a predictor for osteogenic differentiation or VC of VSMCs. Moreover, adiponectin administration reduced the expression of Runx2, calcium deposition and mineralization in smooth muscle cells (Lu et al. 2015). We found that β-GP significantly induced the expression of Runx2 in VSMCs, which was reversed by gAd treatment. As an important member of lipid metabolism, the gAd-mediated improvement of VC may be accomplished through regulating the homeostasis of lipid metabolic pathway. Additionally, compared with full-length adiponectin, gAd, a globular domain of full-length adiponectin, is more effective in improving insulin resistance and increasing fatty acid oxidation (Yamauchi et al. 2001; Berg et al. 2001; Fruebis et al. 2001; Yamauchi et al. 2002; Goldfine and Kahn 2003). Our data further clarified the significant alleviatory effects of gAd on VC in VSMCs in vitro and in uremic rats in vivo.

The Wnt/β-catenin pathway has been demonstrated to promote osteogenic differentiation of bone mesenchymal stem cell (BMSC) (Cawthorn et al. 2012; Qiu et al. 2007; Si et al. 2006). Adiponectin regulates BMSCs osteogenic differentiation and osteogenesis through enhancing the activation of β-catenin (Wang et al. 2017). Adiponectin also impedes Wnt/β-catenin-signaling pathways by regulating the expression of WIF1 in human breast carcinoma cells (Liu et al. 2008). In addition, adiponectin increases the expression of phosphorylated AKT and p65 in osteoblastic cells (Su et al. 2015). These studies suggest that adiponectin-mediated the changes of osteogenic differentiation has the involvement of Wnt/β-catenin and PI3K/AKT pathway. This study found that gAd inhibited VC of VMSCs along with the inhibition of PI3K/AKT and Wnt/β-catenin pathway. And the activation of the two signaling pathway significantly abolished the improvement of gAd. Of note, Wnt/β-catenin pathway should play a dominant role in this process. Thus, it further confirmed that Wnt/β-catenin pathway may be required for VC in VSMCs.

Runx2, a downstream component of the Wnt/β-catenin signaling pathway, is the master transcription factor required in determining the osteoblastic lineage (Stains and Civitelli 2003; Krane 2005). The nephroblastoma overexpressed protein promotes the expression of Runx2 in osteoblasts via inhibiting AKT expression (Chen et al. 2019). The activation of Wnt/β-catenin signaling promotes VC via directly modulating Runx2 in VSMCs (Cai et al. 2016). Increased osteogenic transformation of isocoumarin A is associate with the activation of the PI3K-AKT cascade-activated BMP/Runx2 signaling pathway (Liu et al. 2019a). The above studies show that the expression of Runx2 is closely related with Wnt/β-catenin and PI3K/AKT signaling pathway during osteogenic differentiation or VC. In the present study, our results showed that gAd significantly inhibited the expression and activation of nuclear Runx2 in β-GP-induced VSMCs, whereas the gAd-mediated improvement on VC and activation of Runx2 in β-GP-induced VSMCs were observably abolished by the administration of agonists of Wnt and/or AKT. These results indicated that gAd could reduce calcification of VSMCs by decreasing the expression of Runx2, partly through regulating PI3K/AKT and Wnt/β-catenin-signaling pathways. However, it is unclear whether these two signals act alone or interact on each other during the regulatory process of VC induced by gAd. In addition, as a nuclear transcription factor, the elevation of Runx2 activity regulated by PI3K/AKT might involve the nuclear regulatory molecules, such as nuclear β-catenin. The activation of Wnt/β-catenin is accompanied by the enhancement of nuclear β-catenin activity. However, the mechanism by which nuclear β-catenin regulates the transcriptional activity of Runx2 needs further investigation.

VC in uremic patients is related to abnormal lipid metabolism (Opdebeeck et al. 2020). Uremia patient with VC disease always present disorder of lipid metabolism, hypertriglyceridemia and low levels of HDL (Chan 1990). The regulation of Wnt/β-catenin such as Wnt1, Wnt4 and β-catenin and PI3K/AKT pathways by adiponectin contributes to the improvement of VC in patients with uremia (Ponnusamy et al. 2018; He et al. 2019). In the present study, gAd inhibited calcium nodules in a rat model of uremia, along with the inhibition of Wnt/β-catenin. Combined with the in vitro assay, we speculated that PI3K/AKT signaling pathway might be also inhibited accompanied by the injection of gAd. Possibly, gAd ameliorated VC in uremic rat depending on the inactivation of PI3K/AKT and Wnt/β-catenin pathway. In CKD patients, adiponectin improves the symptoms of CKD patients by increasing the level of HDL and decreasing the content of triglyceride (Yanai and Yoshida 2019). Therefore, we consider that gAd would balance the homeostasis of lipid metabolism of VSMCs, and subsequent the improvement of VC in uremia patients, possibly by reducing nuclear Runx2 activity via PI3k/AKT and Wnt/β-catenin signaling pathways.

## Conclusion

In summary, this study indicated that gAd protected VSMCs from calcification. It also showed that gAd could mitigate calcium nodules formation in aortic vessels of uremic rat. Mechanistically, gAd inhibited osteoblastic differentiation of VSMCs via declining the activation of the PI3K/AKT and Wnt/β-catenin pathways and nuclear transcription factor Runx2. Possibly, gAd might be one of the potential candidates for clinical treatment of VC in uremia patients.

## Supplementary Information

Below is the link to the electronic supplementary material.

Supplementary file1 (DOCX 414 kb)

## Data Availability

All data generated or analysed during this study are included in this published article.
