# Supplementary material for: Globular adiponectin inhibits osteoblastic differentiation of vascular smooth muscle cells through the PI3K/AKT and Wnt/β-catenin pathway
Source: J Mol Histol. 2021 Aug 16;52(5):1067–80. doi: 10.1007/s10735-021-10012-2 (PMC8487883; doi:10.1007/s10735-021-10012-2)
Supplement: Supplementary file 1 — Supplementary file1 (DOCX 414 kb) [file 10735_2021_10012_MOESM1_ESM.docx]

**Supplemental Fig. 1 The morphology and purity of VSMCs. (a)** The morphology of VSMCs was observed by an inverted microscope. **(b)** Detection of the expression of α-SMA in VSMCs by immunofluorescence. α-SMA: actin alpha, smooth muscle aorta; Nuclei, 4’,6-diamidino-2-phenylindole (DAPI, blue).

**Supplemental Fig. 2** **Effects of gAd on osteoblastic differentiation of human VSMCs. (a)** Detection of Runx2 subcellular localization using immunofluorescence. The nucleus was stained with Hoechst (blue) and Runx2 by antibody (red). Scale bar: 20 μm. **(b)** Western blot showed that the expression of WIF-1, phosphorylation-β-catenin, β-catenin, Runx2, phosphorylation-AKT, AKT, BMP2, ALP, phosphorylation-STAT3 and STAT3 protein in VSMCs treated by different chemical compound. **(c)** Quantitative analysis of protein expression of Runx2, WIF-1, BMP2, ALP, phosphorylation-β-catenin/β-catenin, phosphorylation-AKT/AKT, phosphorylation-STAT3/STAT3. The data are shown as the means±SD. N=3 per group.^*^*P*＜0.05, ^**^*P*＜0.01.β-Gp: β-glycerol phosphate; gAd: globular adiponectin.
